# Crosstalk between ferroptosis and innate immune in diabetic kidney disease: mechanisms and therapeutic implications

**DOI:** 10.3389/fimmu.2025.1505794

**Published:** 2025-02-28

**Authors:** Jinyang Wang, Haonan Shi, Ye Yang, Xueli Gong

**Affiliations:** ^1^ Department of Geriatric Integrative, Second Affiliated Hospital of Xinjiang Medical University, Urumqi, Xinjiang, China; ^2^ School of Medicine, Shanghai University, Shanghai, China; ^3^ Department of Pathophysiology, School of Basic Medical Science, Xinjiang Medical University, Urumqi, Xinjiang, China

**Keywords:** diabetic kidney disease, ferroptosis, innate immune, inflammation, therapeutic implications

## Abstract

Diabetic kidney disease (DKD) is a prevalent complication of diabetes mellitus (DM), and its incidence is increasing alongside the number of diabetes cases. Effective treatment and long-term management of DKD present significant challenges; thus, a deeper understanding of its pathogenesis is essential to address this issue. Chronic inflammation and abnormal cell death in the kidney closely associate with DKD development. Recently, there has been considerable attention focused on immune cell infiltration into renal tissues and its inflammatory response’s role in disease progression. Concurrently, ferroptosis—a novel form of cell death—has emerged as a critical factor in DKD pathogenesis, leading to increased glomerular filtration permeability, proteinuria, tubular injury, interstitial fibrosis, and other pathological processes. The cardiorenal benefits of SGLT2 inhibitors (SGLT2-i) in DKD patients have been demonstrated through numerous large clinical trials. Moreover, further exploratory experiments indicate these drugs may ameliorate serum and urinary markers of inflammation, such as TNF-α, and inhibit ferroptosis in DKD models. Consequently, investigating the interplay between ferroptosis and innate immune and inflammatory responses in DKD is essential for guiding future drug development. This review presents an overview of ferroptosis within the context of DKD, beginning with its core mechanisms and delving into its potential roles in DKD progression. We will also analyze how aberrant innate immune cells, molecules, and signaling pathways contribute to disease progression. Finally, we discuss the interactions between ferroptosis and immune responses, as well as targeted therapeutic agents, based on current evidence. By analyzing the interplay between ferroptosis and innate immunity alongside its inflammatory responses in DKD, we aim to provide insights for clinical management and drug development in this area.

## Highlights

- Hyperglycemia and chronic inflammation contribute to Ferroptosis by inducing iron overload, compromising the antioxidant system, and promoting lipid peroxidation, thereby playing a pivotal role in DKD progression.
- Innate immunity, inflammatory responses, and oxidative stress often coexist. Renal inflammatory cell infiltration and its phenotypic transitions during DKD are crucial to disease progression.
- Renal resident and immune cells engage in a complex interaction between ferroptosis and inflammatory responses in DKD, with Nrf2 signaling pathway serves as a bidirectional bridge.
- SGLT2i benefit multiple organs and effectively mitigate renal inflammation and ferroptosis. Targeting both the inflammatory response and ferroptosis simultaneously may represent a promising direction for DKD drug development.

## Open question

What experimental evidence and molecular mechanisms demonstrate how ferroptosis and innate immune-inflammatory responses contribute to disease progression in DKD?

How do ferroptosis and innate immune responses interact each other within the context of DKD?

How the interaction model between ferroptosis and inflammation to guide therapeutic agent development for DKD?

## 1 Introduction

Diabetic kidney disease (DKD) is a prevalent microvascular complication of diabetes mellitus (DM) (1, 2) that has garnered increasing attention as the global incidence of DM rises. DKD is characterized by persistent proteinuria and progressive renal function loss (3). Its pathogenesis is multifaceted, involving hemodynamic disturbances, chronic inflammatory injury, and the accumulation of advanced glycation end-products (AGEs) (4). Immune cell infiltration and aseptic inflammatory injury in renal tissue significantly contribute to DKD progression, with the innate immune response being particularly relevant (5). Under persistent hyperglycemia, hypoxia, and the accumulation of AGEs (6), innate immunity initiates early renal inflammatory injury in DKD by recruiting and polarizing macrophages to the M1 phenotype, along with the secretion of pro-inflammatory factors, including IL-1β, IL-6, and TNF-α. This disruption of the charge barrier of glomerular filtration membranes (7) results in persistent proteinuria and tubular injury. Macrophage conversion to the M2 anti-inflammatory phenotype and the subsequent release of inflammatory factors, such as IL-10 and TGF-β, play a crucial role in the advancement of renal fibrosis in DKD (8–10). This progression includes glomerular mesangial cell proliferation and interstitial fibrosis (11), which ultimately leads to significantly reduced estimated glomerular filtration rate (eGFR) and the necessity for renal replacement therapy. Macrophage-myofibroblast transition (MMT) is recognized as a hallmark of rapid renal fibrosis progression in DKD (6). However, pro-inflammatory and pro-fibrotic processes mediated by immune cells infiltrating the kidneys often coexist and interact. Key pathways involved in regulating renal inflammatory injury in DKD include the JAK-STAT pathway, the TLR4 pathway, the NLR3-inflammatory vesicle pathway, and the TGF-β1/Smad pathway (5, 12). Targeting specific inflammatory cells, immune molecules, or regulatory pathways holds promise for ameliorating renal injury in DKD (13).

The cellular mechanisms underlying the development of DKD involve endoplasmic reticulum stress, mitochondrial damage, and dysregulated cell death (14–16). Among these, cell death is a crucial mechanism in DKD progression, enhancing our understanding of its pathogenesis as new forms of cell death are discovered. One such form, known as ferroptosis, is a recently identified type of non-apoptotic cell death characterized by the accumulation of lethal, iron-dependent lipid peroxides (17). In pathological states, iron overload, resulting from disrupted intra- and extracellular iron metabolism, is a hallmark of ferroptosis (18). Additionally, lipid remodeling promotes the synthesis of unsaturated fatty acids (19), with an increased proportion of unsaturated fatty acid phospholipids further facilitating ferroptosis. Importantly, in DKD, downregulation of GPx4 and xCT system expression, along with decreased activity in response to stimuli such as high glucose, hypoxia, pro-inflammatory cytokines, and damage-associated molecular patterns (DAMP), constitutes a critical step in the progression of ferroptosis (20–22). The molecular mechanisms of ferroptosis involve several signaling pathways, including the IRP-IRE pathway, Nrf2 pathway, p53 pathway, and STAT (23). The role of ferroptosis in the pathogenesis of DKD is a significant focus of research. By targeting iron production, ferroptosis can inhibit mesangial cell proliferation and tubular injury while improving renal interstitial fibrosis (24). Evidence from clinical trials and animal studies investigating the therapeutic effects of related drugs on DKD indicates that ameliorating the renal inflammatory response and cellular ferroptosis is vital for delaying DKD progression (25, 26). Furthermore, the combined targeting of the innate immune response and ferroptosis may lead to innovative approaches in drug research and development for DKD.

Currently, research on the mechanisms of immune infiltration and ferroptosis in DKD, along with their interactions and the availability of targeted therapies, is evolving rapidly. Existing reviews struggle to provide a comprehensive overview of these dynamics. This paper aims to explore the interactions between ferroptosis and innate immune infiltration in DKD pathogenesis, focusing on core mechanisms that drive disease progression. The goal is to offer new insights for future research in this area.

## 2 Core mechanisms of ferroptosis

### 2.1 Iron overload

**Figure 1:**
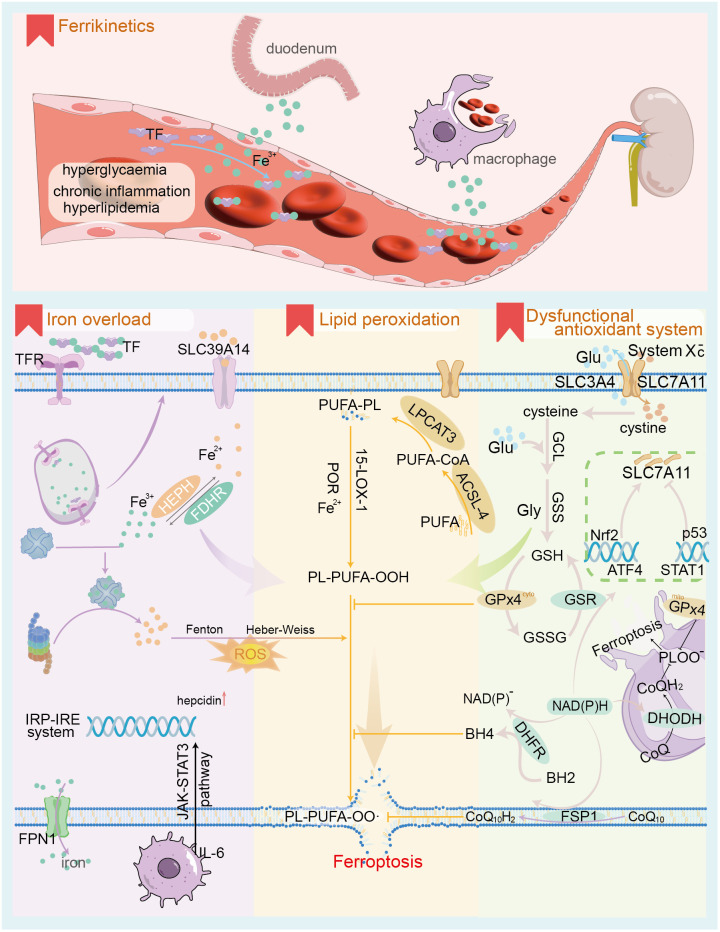
The core mechanism of ferroptosis. The upper panel illustrates the characteristics of iron ion dynamics in the context of DKD; the lower panel depicts the 3 core mechanisms of ferroptosis, namely iron ion overload, antioxidant system dysfunction and lipid peroxidation. TF, transferrin; TFR, transferrin receptor; SLC39A14, solute carrier family 39 member 14 (as known as ZIP14); FPN1, ferroportin1; IRP-IRE, Iron regulatory protein-iron responsive element; ROS, reactive oxygen species; PUFA, polyunsaturated fatty acid; ACSL-4, Acyl-CoA Synthetase Long Chain Family Member 4; LPCAT3, Lysophosphatidylcholine acyltransferases3; 15-LOX-1, 15-lipoxygenase-1; POR, cytochrome p450 oxidoreductase; Glu, glutamate; GCL, Glutamate cysteine ligase; GSS, glutathione synthetase; GSH, glutathione; GPx4, Glutathione Peroxidase 4; GSSG, oxidized glutathione; GSR, glutathione reductase; SLC7A11, Solute Carrier Family 7 Member 11; Nrf2, Nuclear factor erythroid 2-related factor 2; ATF4, activating transcription factor 4; STAT1, signal transducer and activator of transcription 1; CoQ, ubiquinone; DHODH, Dihydroorotate Dehydrogenase; DHFR, dihydrofolate reductase; BH4, tetrahydrobiopterin; FSP1, Ferroptosis suppressor protein 1.

Intracellular iron overload is a distinguishing characteristic of ferroptosis compared to other forms of cell death (**Figure 1**). Under normal conditions, iron homeostasis in the human body is maintained by a complex regulatory mechanism involving absorption, transport, storage, utilization, and recycling of iron. Iron sources are categorized into exogenous and endogenous pathways. Exogenous sources involve dietary iron reduction and its transport into duodenal epithelial cells via divalent metal transporter 1 (DMT1) (27), which includes four isoforms, notably DMT1A-I, predominantly expressed in the duodenum and kidney. Fe^2+^ entering intestinal epithelial cells is transported into the bloodstream via Ferroportin 1 (FPN1) at the basement membrane, contributing to 5-10% of the body’s iron. This process is regulated by hepcidin, a peptide synthesized and secreted by the liver. Hepcidin binds to FPN1, facilitating its endocytotic degradation and inhibiting iron absorption in the small intestine (28). The endogenous pathway derives from the degradation of senescent erythrocytes by macrophages for iron recycling, accounting for approximately 90% of the body’s iron supply. Iron absorbed into the bloodstream is oxidized and primarily bound to transferrin (TF), which transports it to iron-demanding organs such as the bone marrow (29). Insufficient iron supply leads to iron deficiency diseases, while iron overload elevates levels of non-transferrin bound iron (NTBI), resulting in oxidative damage to cells. NTBI is a reactive iron species readily taken up by parenchymal cells, such as hepatocytes, leading to intracellular iron overload. This occurs via the Fenton reaction (H_2_O_2_ + Fe^2+^ —> Fe^3+^ + OH**·** + OH^-^; Fe^3+^ + H_2_O_2_ —> Fe^2+^ + O_2_ + 2H^+^; Fe^2+^ + O_2_ —> Fe^3+^ + O_2_ **·** ^-^) and the Haber-Weiss reaction (iron-catalyzed O_2_ **·** ^-^ + H_2_O_2_ —> OH^-^ + O_2_ + OH**·)**, generating reactive oxygen species (ROS) that damage cell membranes, organelles, and DNA (30, 31).

The transferrin receptor (TFR) is the primary receptor mediating TF access to most cells (32). TFR-mediated TF endocytosis leads to the release of Fe²^+^ from vesicles containing the TFR-TF complex in acidic environments, while unloaded TFR-TF returns to the cell membrane for reuse. Exocytosis of intracellular iron, reliant on FPN1, also known as solute carrier family 40 member 1 (SLC40A1), is the only mechanism capable of transporting non-heme iron out of the cell (33). Changes in the quantity, structure, and activity of both TFR and FPN1 can disrupt iron homeostasis, contributing significantly to ferroptosis. Besides its physiological roles in synthesizing hemoglobin, iron-containing proteins, and enzymes, excess iron entering the cell is stored by ferritin, which can hold approximately 4,500 Fe³^+^ ions. Thus, ferritin is crucial for maintaining intracellular iron homeostasis (34). Iron overload in tissues results in the conversion of intracellular ferritin to hemosiderin (35). Iron homeostasis is achieved through the interaction of iron regulatory proteins (IRPs) and target gene sequences, known as iron response elements (IREs). Notably, type 1 IRP (IRP1) operates independently of IRE, exerting cis-aconitase activity in the cytosol (36). As intracellular iron levels decline, the iron at position 4 in the iron-sulfur cluster of IRP1 detaches, leading to disassembly of the cluster. IRP1 binds to the IRE, enhancing mRNA stability, which upregulates the expression of DMT1 and TFR, thereby facilitating iron uptake. Conversely, high intracellular iron levels downregulate this process. Furthermore, the inflammatory factor IL-6 upregulates hepcidin expression and downregulates FPN1 via the JAK-STAT3 and SMAD signaling pathways (28), which is essential for immune cell function. In contrast, hypoxia and anemia stimulate erythropoietin (EPO) production, which contributes to the synthesis and secretion of erythroferrone by nucleated erythrocytes, effectively downregulating hepcidin expression (37). The accumulation of ROSs resulting from intracellular iron overload initiates ferroptosis.

### 2.2 Dysfunctional antioxidant systems

Antioxidant system dysfunction is a critical prerequisite to ferroptosis. Ferroptosis is primarily triggered by increased ROS production mediated by iron, coupled with insufficient clearance, resulting in lethal lipid peroxidation and subsequent cell death. The antioxidant system associated with ferroptosis comprises the glutathione peroxidase 4 (GPx4) regulatory system and GPx4-independent pathways (38). These include the ferroptosis suppressor protein 1-coenzyme Q10 (FSP1-CoQ10) pathway at the cell membrane (39), the GTP cyclohydroxylase1-tetrahydrobiopterin (GCH1-BH4) pathway in the cytoplasm (40), and the dihydroorotate dehydrogenase (DHODH-CoQH2) pathway in mitochondria (41). Glutathione peroxidase 4 (GPx4), located in the cytoplasm and mitochondria, reduces lipid peroxidation products generated from ROS-induced polyunsaturated fatty acids (PUFA) to lipids and alcohols, utilizing glutathione (GSH) as a reducing cofactor (38). The catalytic process follows a ‘ping-pong mechanism’. The GPx4 system represents a classical pathway for resistance to ferroptosis, requiring adequate GSH and functional GPx4 enzymes for proper operation (42). GPx4 belongs to the selenoprotein family; thus, selenium excess or deficiency impacts its expression and activity. Consequently, lipid peroxide accumulation triggers iron-dependent, non-apoptotic cell death, termed ferroptosis (43). Intracellular GSH biosynthesis relies on the cystine/glutamate antiporter system (System Xc -) located at the cell membrane, composed of the functional subunit SLC7A11 (also known as xCT) and the regulatory subunit SLC3A2. This system transfers cystine into the cell at a 1:1 ratio while simultaneously excreting glutamate (44). Cysteine entering the cell is oxidized and subsequently synthesized into GSH via glutamate cysteine ligase (GCL) and glutathione synthase (GSS), utilizing NADPH as a reducing factor.

The expression level of SLC7A11 significantly influences the activity of the Xc^-^ system and the cellular uptake of cystine. Regulation of SLC7A11 occurs at both the mRNA and protein levels, primarily involving the transcription factors nuclear factor erythroid 2-related factor 2 (Nrf2) and activating transcription factor 4 (ATF4), which positively regulate SLC7A11 (45, 46). In contrast, transcription factors such as p53 and signal transducer and activator of transcription 1 (STAT1) negatively regulate SLC7A11 (47, 48). Under oxidative stress, the E3 ubiquitin ligase kelch-like ECH-associated protein 1 (KEAP1) inhibits the degradation of Nrf2 proteins. Consequently, Nrf2 translocate into the nucleus, where it binds to the antioxidant response element (ARE) in the promoter regions of various antioxidant-related genes. This interaction upregulates SLC7A11 expression and enhances cellular antioxidant capacity by promoting GPx4 expression (45). Likewise, ATF4 plays a crucial role in regulating amino acid metabolism, endoplasmic reticulum stress, and redox homeostasis. Under amino acid starvation, ATF4 mRNA translation is promoted, leading to the nuclear translocation of ATF4 protein. The protein then binds to the amino acid response element in the gene promoter region, enhancing the expression of genes related to amino acid metabolism, including SLC7A11 (46). Elevated expression of SLC7A11 supplies adequate precursors for intracellular GSH synthesis and boosts the antioxidant capacity of cells. STAT1 is a key transcription factor that regulates immune response-related gene expression and mediates the interferon signaling pathway. In an inflammatory state, cytokines such as IFN-γ bind to cell membrane receptors, activating intracellular receptor fragments and recruiting Janus kinases 1 and 2 (JAK1 and JAK2). Activated JAK further catalyzes the recruitment of STAT1 and phosphorylates it, forming a dimer that translocates into the nucleus. Here, it binds to specific sequences in the gene promoter region, initiating downstream transcription and exerting immune regulatory effects. In fibroblasts, IFN-γ-activated STAT1 can inhibit SLC7A11 transcription, reduce intracellular GSH levels (48), and increase sensitivity to ferroptosis. Similarly, activation of STAT3/5 significantly reduces mRNA and protein levels of xCT, thereby negatively regulating SLC7A11 (49). Additionally, epigenetic modifications—including protein acetylation, methylation, and ubiquitination—are also implicated in the regulation of SLC7A11. Regulation of SLC7A11 protein levels encompasses modulation of the subunit SLC3A2, cell membrane adhesion molecules CD44v, and the mammalian target of rapamycin complex 2 (mTORC2). mTORC2 inhibits the cysteine transport activity of SLC7A11 by directly phosphorylating serine 26 at its N-terminus or indirectly through the substrate AKT (50), thereby enhancing sensitivity to ferroptosis.

Beyond the GPx4 pathway, it has been established that certain tumor cells can survive and proliferate despite GPx4 deficiency, owing to compensatory non-GPx4-dependent regulatory pathways. In the FSP1-CoQ10 pathway (39), acylated FSP1 at the cell membrane functions as an oxidoreductase for CoQ10, crucially capturing lipophilic free radicals and inhibiting lipid peroxidation. Additionally, vitamin E aids in regenerating reduced CoQ10H2. Within the GCH1-BH4 pathway (40), GCH1 serves as a key enzyme in the *de novo* synthesis of BH4. In the phospholipid bilayer, BH4 captures lipid-derived peroxyl radicals, terminating lipid peroxidation while maintaining ferroptosis resistance by regenerating BH4 via dihydrofolate reductase (DHFR). The DHODH-CoQH2 pathway is crucial for managing oxidative stress within mitochondria (41). Dihydroorotate dehydrogenase (DHODH) primarily catalyzes the fourth step of pyrimidine nucleotide synthesis, oxidizing dihydroorotic acid (DHO) to orotic acid while transferring electrons to ubiquinone in the mitochondrial inner membrane. This process reduces ubiquinone to dihydroubiquinone (CoQH2), facilitating the capture and clearance of lipid peroxides. Adverse conditions causing dysfunction of the lipid peroxide-associated antioxidant system, especially the GPx4 pathway, result in lipid peroxide accumulation and promote ferroptosis.

### 2.3 Lipid peroxidation

Specific lipid peroxides and peroxide free radicals containing polyunsaturated fatty acid (PUFA) chains serve as the “executing molecules” of cellular ferroptosis (51), influencing the fluidity, integrity, and stability of both cellular and organelle membranes. Notably, numerous studies indicate that omega-3 PUFA, including alpha-linolenic acid and docosapentaenoic acid, exert anti-inflammatory effects, whereas omega-6 PUFA, such as linoleic acid, adrenic acid, and arachidonic acid, promote inflammation (52, 53). The phospholipids specifically involved in ferroptosis lipid peroxidation are phosphatidylethanolamine (PE) containing arachidonic acid (AA) or adrenic acid (AdA), referred to as PE-AA and PE-AdA (38). Long-chain acyl-CoA synthase 4 (ACSL4) catalyzes the reaction between AA and CoA, yielding the intermediate product AA-CoA. This process consumes ATP and represents the rate-limiting step. AA-CoA can gain two carbons through the action of fatty acid elongase to form AdA-CoA (54). Although AA concentrations at the cell membrane are low compared to AdA, PE-AA levels in ferroptosis cells are significantly higher than those of PE-AdA. This indicates that ACSL4 exhibits greater specificity and affinity for AA, leading cells to preferentially utilize AA for phospholipid synthesis (55). Subsequently, lysophosphatidylcholine acyltransferases 3 (LPCAT3) catalyzes the esterification of AA-CoA and AdA-CoA with the fatty chain of PE, resulting in the precursors of ferroptosis, PE-AA and PE-AdA. These specific unsaturated phospholipids can generate PE-AA-OOH and PE-AdA-OOH through either non-enzymatic self-oxidation or enzymatic oxidation. It is widely accepted that non-enzymatic self-oxidation of phospholipids results from hydroxyl radicals produced by the Fenton reaction, which attack PUFA (56). Enzymatic oxidation of phospholipids involves the cooperation of 15-lipoxygenase (15-LOX) and cytochrome P450 oxidoreductase (57). Specific lipid peroxides can be captured and reduced by antioxidants, including GPx4, CoQ10H2, and BH4 (58).

The mechanisms by which specific lipid peroxides and peroxidation-free radicals induce ferroptosis are categorized into direct and indirect damage effects (17). Direct damage involves the accumulation of lipid peroxides in both cell and organelle membranes, leading to alterations in membrane permeability and integrity, which can ultimately compromise the proteins and biomolecules embedded within the phospholipid bilayer. In contrast, the indirect damage mechanism is mediated by degradation products of lipid peroxides, including malondialdehyde (MDA) and 4-hydroxynonenal (4-HNE), which can adversely affect vital macromolecules such as proteins and DNA within cells. Notably, key markers of ferroptosis include mitochondrial fragmentation, increased membrane density, and decreased cristae, accompanied by no significant changes in nuclear morphology. However, the precise mechanisms through which specific lipid peroxides induce mitochondrial alterations remain to be elucidated.

PUFA serves as a crucial substrate for ferroptosis, with cell membrane phospholipids containing significant amounts of PUFA, facilitating the formation of lipid peroxides. Consequently, factors influencing lipid remodeling and increasing cellular PUFA levels can heighten sensitivity to ferroptosis. Three pivotal pathways influencing lipid remodeling are mediated by phospholipase, the E3 ubiquitin ligase MDM2/MDMX complex, and energy stress (59–61). Energy stress occurs when glucose supply is inadequate, leading to decreased ATP concentrations and an increased AMP/ATP ratio. This activates AMP-dependent protein kinase (AMPK), which regulates various ATP-utilizing metabolic pathways, including fatty acid synthesis. Activated AMPK inhibits PUFA synthesis by blocking acetyl-CoA carboxylase (61), thus affecting lipid peroxide generation and decreasing cellular sensitivity to ferroptosis.

## 3 Ferroptosis and DKD

### 3.1 Disordered iron metabolism

In diabetic kidney disease (DKD), the kinetic state of iron is altered, leading to their redistribution (62) (**Figure 1**). Patients with DKD frequently experience anemia, primarily due to iron deficiency, which comprises both absolute and relative forms (63). The latter occurs when circulating blood iron is deficient despite adequate total body iron content and is particularly prevalent in DKD. Contributing factors include chronic inflammation, reduced synthesis and secretion of EPO, and diminished tissue sensitivity to EPO (64, 65). This cascade results in increased hepatic hepcidin secretion and downregulation of FPN1 levels. Simultaneously, the destruction of renal units and progressive loss of renal function lead to reduced EPO production, downregulating signals that stimulate proerythrocyte proliferation and subsequently decreasing bone marrow hematopoiesis. Observational studies indicate that, alongside a significant reduction in blood iron levels, renal iron levels are markedly elevated in DKD patients (66). Furthermore, a strong correlation between urinary albumin and TF levels suggests that this phenomenon may be due to the effects of proteinuria. Since the isoelectric point of albumin (PI=4.9) is notably lower than that of transferrin (PI=5.7), this disruption of the glomerular filtration membrane, coupled with decreased selectivity of the charge barrier for permeable substances, likely explains the observed correlation between albumin and TF levels in urine. Further studies reveal TF accumulation in the cytoplasm of glomerular podocytes in end-stage DKD (67). The iron ions released by TF exacerbate cellular oxidative stress and insulin resistance in patients with type 2 diabetes mellitus (T2DM). Additionally, urinary TF levels correlate with progressive renal lesions, including interstitial fibrosis, renal tubular cell atrophy, and infiltration of inflammatory cells in the renal interstitial (68). Consequently, in the context of DKD, glomerular filtration of TF increases while reuptake decreases, resulting in the accumulation of iron ions within the renal tubules. Hydroxyl radicals are generated through the Fenton and Haber-Weiss reactions, inducing oxidative stress damage in the renal tubules. Limiting iron intake in T2DM rats has been shown to reduce ROS generation, helping to prevent the onset of early kidney disease (69). Further research is required to explore additional sources of iron that may play a role in DKD.

Dysregulation of renal iron homeostasis in DKD is evidenced by increased cellular uptake and decreased excretion of iron (70). ZRT/IRT-like protein 14 (ZIP14), a membrane transporter that mediates the cellular uptake of iron, zinc, and other ions, exhibits elevated expression in renal tissues from both DKD patients and a rat model of DKD (71). This upregulation is associated with intracellular iron deposition and oxidative damage. Notably, silencing ZIP14 expression with siRNA inhibited ferroptosis in high glucose-treated human renal proximal tubule cells (HK2) (71). In chronic kidney disease (CKD), the expression of transporter proteins responsible for iron ion transfer to renal tubules, including ZIP8, DMT1, and ZIP14, is elevated (70). This increase, coupled with enhanced expression of iron storage proteins and reduced levels of FPN1, facilitates intracellular iron accumulation and ferroptosis. Mice with specific knockout of FPN1 in renal proximal tubules show substantial iron accumulation without notable alterations in renal function. However, they exhibit more severe renal injury and ferroptosis when subjected to acute kidney injury (72). This evidence indicates that renal iron accumulation heightens susceptibility to ferroptosis, particularly when combined with adverse stimuli such as hyperglycemia and chronic inflammation. Recent studies show that the Stimulator of Interferon Genes (STING) is aberrantly activated in DKD. Knockdown of STING mitigates renal pathological injury by suppressing ferroptosis (73). As a transmembrane protein located on the endoplasmic reticulum, STING is closely linked to innate immunity, inflammatory responses, and energy metabolism (74). Mechanistically, upon receiving external stimuli, STING dissociates from the endoplasmic reticulum membrane and becomes activated. Activated STING phosphorylates interferon regulatory factor 3 (IRF3), facilitating its transfer to the nucleus and enhancing the expression of inflammatory factors, which leads to inflammatory damage. Moreover, inhibiting STING reduces the degradation of ubiquitinated FPN1 by proteasomes, stabilizing FPN1 protein levels and subsequently mitigating ferroptosis. STING’s regulatory role in FPN1 presents a novel avenue for understanding ferroptosis (73).

Ferritin in vertebrates is composed of heavy (FtH) and light (FtL) chains. The FtH chain is catalytically active, rapidly converting Fe²^+^ to Fe³^+^, while the FtL chain primarily facilitates the aggregation of iron ions into nuclei (75). Tissues involved in iron storage exhibit a higher FtL specific gravity; however, renal tissue has a greater FtH specific gravity, indicating a reduced capacity for iron storage (76). In the kidney, the proximal tubule expresses high levels of both FtH and FtL. Additionally, the apical membrane of the proximal tubule contains TFR1 and megalin/cubilin receptors (77), which mediate the reabsorption of transferrin-bound iron and hematogenous iron, respectively. Furthermore, FPN1 on the basolateral side of the membrane recycles iron reabsorbed from the proximal tubule into circulation (78). Consequently, the proximal tubule serves as a central site for urinary iron reabsorption and is particularly sensitive to ferroptosis activators (79, 80). Renal iron overload in the context of DKD can be attributed to three primary factors. First, an increase in the glomerular filtration of iron ions and iron-containing proteins, coupled with reduced iron reabsorption and the infiltration of inflammatory cells, leads to excess iron accumulation in renal tissues. Second, a rise in membrane proteins that transport iron ions into cells, combined with a decrease in FPN1, exacerbates cellular iron overload in renal tissues. Finally, renal intracellular ferritin, with a limited capacity to sequester iron, is more susceptible to ferroptosis.

### 3.2 Depressed lipid antioxidant system

In the context of diabetic kidney disease (DKD), the antioxidant system associated with ferroptosis is impaired. Observational studies indicate that patients with CKD and end-stage renal disease (ESRD) frequently present with selenium deficiency (81–83). A selenium-rich diet can significantly reduce the risk of CKD (84). Randomized double-blind trials demonstrate that selenium supplementation significantly reduces IL-6 and MDA levels, the latter being a biomarker of lipid peroxidation associated with ferroptosis (83). This evidence suggests that selenium supplementation can inhibit ferroptosis and reduce inflammation. Mechanistically, selenocysteine contains selenium and is integral to the catalytic active site of GPx4; adequate selenium ensures its activity. Absence of selenium in GPx4 renders it inactive, rendering cells highly sensitive to peroxide-induced ferroptosis (85). A recent cohort study confirmed this relationship, finding that GPx4 expression in the kidney tissue of DKD patients was significantly reduced; GPx4 levels correlated strongly with proteinuria and the urinary albumin-to-creatinine ratio (UACR). Moreover, DKD patients exhibiting low GPx4 levels demonstrated a substantially increased risk of developing ESRD during follow-up (86). Paradoxically, a recent bidirectional Mendelian randomization study suggested that elevated selenium levels may contribute to CKD (87). Therefore, further investigation is warranted to clarify the relationship between DKD and selenium.

Numerous studies have shown that Nrf2 is suppressed in DKD patients (88). Nrf2 is crucial for resistance to oxidative stress; its suppression may be linked to the activation of high mobility group protein 1 (HMGB1), a non-histone DNA-binding protein that regulates nucleosome function and intranuclear transcription while functioning as a DAMP. HMGB1 is closely associated with systemic inflammation and renal injury (89, 90). Recent studies indicate that serum and urine levels of HMGB1 are significantly elevated in DKD patients compared to those with T2DM without nephropathy and are closely associated with TNF receptor superfamily member-1A (TNFR-1) (89). Mechanistically, HMGB1 can bind to receptors for advanced glycation end-products (RAGE) or Toll-like receptors (TLRs), activating the MAPK-NF-κB pathway and contributing to the expression of inflammatory factors (91). This binding also inhibits Nrf2 expression, downregulates SLC7A11 levels, and promotes ferroptosis. Conversely, knockdown of HMGB1 inhibited high glucose-mediated activation of the TLR4/NF-κB axis, resulting in upregulated Nrf2 expression (20) and enhanced cellular antioxidant capacity. Notably, elevated ATF4 expression in DKD mice resulted in severe renal injury by decreasing cellular autophagic flux and increasing collagen type IV levels (21). In DKD, the activation of STING promotes ferroptosis through interaction with GPx4 (22, 92). Paradoxically, activation of the cGAS-STING signaling pathway is dependent on GPx4 activity (92). However, epidemiological evidence indicates that GPx4 levels are significantly reduced in DKD patients (86). Therefore, further investigation is warranted into the subtle relationship between the STING pathway and GPx4 in renal tissues within the context of DKD.

Patients with DKD display significantly elevated levels of p53 and STAT1 (93, 94), which inhibit xCT expression. The downregulation of the Xc^–^ system leads to decreased cystine uptake and GSH synthesis, activates ferroptosis, and promotes the progression of DKD. Elevated expression of transforming growth factor-β1 (TGF-β1) significantly contributes to renal injury in diabetes. By activating both canonical (Smad-dependent) and non-canonical (non-Smad-dependent) signaling pathways (95), TGF-β1 stimulates myofibroblast activation, extracellular matrix (ECM) deposition, mesangial proliferation, and renal fibrosis. Recent studies indicate that TGF-β1 inhibits xCT expression by activating the canonical Smad3 pathway, thereby promoting lipid peroxidation (96). In summary, during DKD, Nrf2 expression is downregulated while p53, STAT1, and TGF-β1 expressions are upregulated. These changes inhibit xCT expression and suppress cellular uptake of cystine and GSH synthesis, significantly reducing cellular antioxidant capacity and promoting the accumulation of ROS and lipid peroxidation. This leads to oxidative stress and cellular ferroptosis. Furthermore, investigating additional ferroptosis antioxidant systems in kidney tissue during DKD, such as FSP1-CoQ10, GCH1-BH4, and DHODH-CoQH2, could provide valuable insights into the occurrence and potential interventions for ferroptosis in the context of DKD.

### 3.3 Lipid remodeling

Glucose metabolism disorders disrupt lipid metabolic homeostasis in renal tissues. A recent study examined the relationship between acetyl-CoA synthetase 2 (ACSS2) and DM renal lipid metabolism, revealing significantly upregulated ACSS2 expression in the renal tissues of both DM patients and DM mice. This upregulation remodels fatty acid metabolism (19) and contributes to mitochondrial oxidative stress by modulating the SIRT1-ChREBP pathway. Chronic inflammatory diseases are associated with an increased ratio of ω-6 to ω-3 PUFAs (97), specifically a relative increase in AA and AdA. Disturbed lipid metabolism, exacerbated by chronic inflammation, is coupled with increased renal expression of ACSL4 due to high glucose stimulation in DKD, mediated by factors such as STING (98, 99). Additionally, renal iron ion overload, resulting from the Fenton and Haber-Weiss reactions, generates hydroxyl radicals that catalyze lipid peroxidation. Consequently, the persistent high glucose and inflammatory stimuli impair the antioxidant system, preventing timely removal of accumulated lipid peroxides. This accumulation promotes ferroptosis and exacerbates renal damage.

## 4 Innate immune and DKD

### 4.1 Intrinsic immune cells

**Figure 2:**
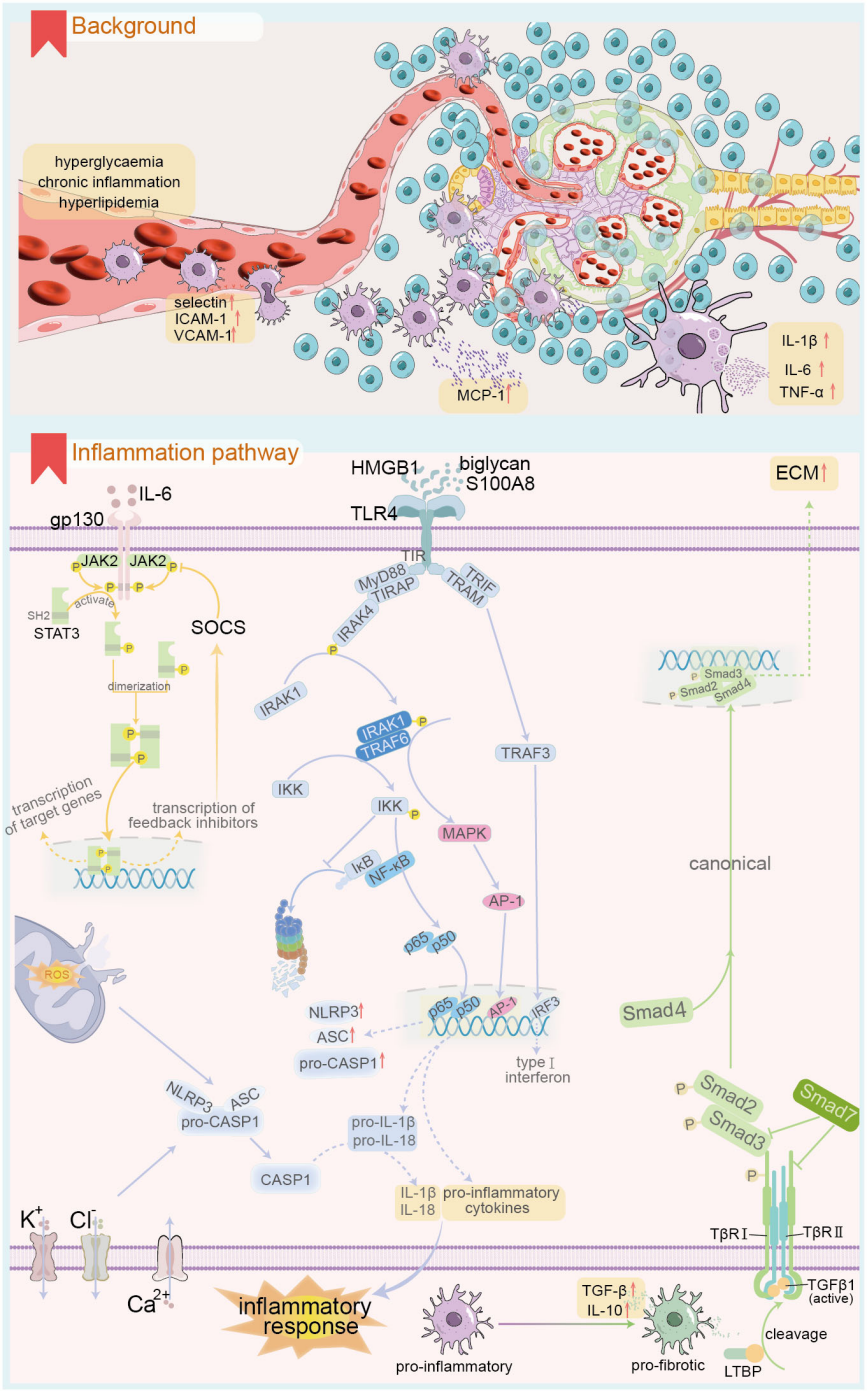
Renal innate immune cell infiltration and related inflammatory response under DKD background. The upper panel demonstrates the renal innate immune cell infiltration in DKD, and the lower panel shows the key signalling pathways involved in the renal inflammatory response in the context of DKD. ICAM-1, intercellular adhesion molecule-1; VACM-1, Vascular Cell Adhesion Molecule-1; MCP-1, Monocyte chemoattractant protein-1; gp130, Glycoprotein 130; STAT, signal transducer and activator of transcription; JAK, Janus kinase; SOCS, suppressors of cytokine signaling; HMGB1, High mobility group box 1; S100A8, S100 calcium binding protein A8; TLR4, Toll like receptor 4; TIR, Toll/interleukin-1 receptor; MyD88, Myeloid differentiation primary response protein 88; TRIAP, TIR-containing adapter protein; IRAK, interleukin-1 receptor-associated kinase; TRIF, TIR Domain-Containing Adaptor-Inducing Interferon-β; TRAM, TRIF-related adaptor molecule; TRAF, TNF receptor-associated factor; IκB, inhibitor of NF-κB; IKK, IκB kinase; MAPK, Mitogen-activated protein kinase; AP-1, activator protein 1; IRF3, interferon-regulatory factor 3; CASP1, Caspase-1; TGF, transforming growth factor; LTBP, TGF-β binding proteins; TβR, TGFβ receptor; ECM, Extracellular matrix.

Innate immune cells are present in blood and tissues and can be categorized into classical innate immune cells stemming from common myeloid precursors in the bone marrow (**Figure 2**), including monocytes, macrophages, mast cells, and neutrophils. Non-classical immune cells, derived from common lymphoid precursors, encompass innate lymphoid cells, such as NK cells, and innate lymphocytes, including NKT and B1 cells (100). Employing single-cell transcriptomics, bioinformatics, and immune infiltration analysis, researchers found that the DKD group exhibited a significant increase in macrophages compared to the healthy control group. Notably, a positive correlation was observed between DKD-related hub genes and innate immune cells, including gamma delta T cells, M2 macrophages, and mast cells, with macrophage infiltration being the most pronounced (101–105). A persistent high-glucose and hypoxic renal microenvironment stimulates the expression of intercellular adhesion molecule-1 (ICAM-1), vascular adhesion molecule-1 (VCAM-1), P-selectin, and L-selectin in renal endothelial cells (106–109). This significantly enhances the adhesion and infiltration of inflammatory cells into renal tissue.

Macrophages play a crucial role in immune infiltration within renal tissue during persistent high glucose exposure (110). Specific inhibition of macrophages can alleviate macrophage-dependent renal inflammation (111, 112). Blood monocytes migrate into renal tissues and differentiate into renal macrophages, exhibiting highly heterogeneous morphologies that vary significantly with the immune microenvironment. In DKD, common stimuli include sustained high glucose, accumulation of AGEs, altered hemodynamics, and the release of DAMPs from cell death (6). Macrophages can be classified into two main types: M1 and M2, based on differences in gene expression and function. M1 macrophages are primarily responsible for the pro-inflammatory response and are involved in the early stages of renal injury in DKD (8). Their polarization can be triggered by pathogen-associated molecular patterns, DAMPs such as S100A9 and IL-1α, as well as pro-inflammatory mediators like IFN-γ and TNF (113). For instance, M1 macrophages can exacerbate high glucose-induced podocyte apoptosis through TNF-α production via the ROS-p38 MAPK pathway. M2 macrophages primarily mediate anti-inflammatory responses (8) and play key roles in repair and fibrosis during the later stages of DKD injury. M2 macrophages can be further subdivided into three distinct subtypes based on phenotype and function: M2a, M2b, and M2c. M2a macrophages are primarily induced by IL-4 and IL-13 (6, 114), facilitating a Th2-like anti-inflammatory immune response, promoting injury repair, and facilitating tissue fibrosis. M2b macrophages are mainly induced by immune complexes, TLRs, and IL-1R ligands, playing a role in immune regulation and activation of Th2-like immune responses. M2c macrophages, induced by IL-10, TGF-β, and glucocorticoids, are associated with immune suppression. Recent advancements in single-cell genomics have opened new avenues for analyzing renal macrophage subtypes (115). T cell immunoglobulin and mucin domain-3 (Tim3) expressed on macrophages is a crucial regulator of the balance between M1 and M2 macrophages, thus modulating pro-inflammatory and anti-inflammatory responses (116, 117). In patients and experimental models of DKD, renal macrophage Tim3 expression is upregulated and positively correlates with renal dysfunction. Mechanistically, Tim3 upregulation activates the NF-κB pathway and TNF-α release, exacerbating inflammatory responses. Notably, specific knockdown of Tim3 ameliorates STZ-induced renal injury (118). The longevity protein Sirt6 plays a role in M1/M2 macrophage transformation. In a DKD rat model, M1 markers like CD86, TNF-α, and inducible nitric oxide synthase (iNOS) were elevated, while Sirt6 expression was significantly reduced, alongside M2 markers such as CD206, IL-4, and IL-10 [REF (119)]. Notably, overexpression of Sirt6 in macrophages enhanced their conversion to the M2 phenotype, ameliorating DKD-associated renal inflammatory injury. Bioactive substances like vitamin D can inhibit M1 macrophage polarization by downregulating the STAT-1-TREM-1 pathway (120). Recent studies have focused on extracellular vesicles rich in leucine-rich alpha-2-glycoprotein 1 (LRG1) as vital communicators between macrophages and resident renal cells, functioning through a TGF-β receptor-dependent mechanism (121). Additionally, extracellular vesicles from macrophages cultured in high glucose can induce mesangial cell proliferation by activating the TGF-β1/Smad3 signaling pathway (122). Furthermore, recent single-cell omics studies indicate that myofibroblasts exhibit the highest ECM gene expression scores among various kidney cell types, suggesting elevated expression of collagen, proteoglycans, and glycoprotein-related genes in these cells (123). Renal macrophages can directly promote the progression of end-stage renal fibrosis in DKD through the MMT. The occurrence of MMT is linked to the M2a phenotype, specifically CD206, and is mediated by the TGF-β1-Smad3 signaling pathway. This transition serves as a hallmark for the progression from chronic kidney inflammation to fibrosis (6).

Mast cells and neutrophils significantly contribute to inflammatory stress in DKD. Cross-sectional studies reveal varying degrees of mast cell infiltration in kidney tissue from DKD patients across nearly all stages (124). The extent of mast cell degranulation correlates positively with tubulointerstitial injury. Anaphylatoxin C3a activates mast cells, directing them toward the inflammatory center and promoting renal fibrosis via the SCF/c-kit signaling pathway (125). The release of chymase, TNF-α, and TGF-β from mast cells exacerbates renal tubulointerstitial injury, with TNF-α most closely linked to DKD progression (124). Long-term administration of chymotrypsin inhibitors in diabetic mice significantly reduces mesangial cell proliferation and improves proteinuria progression (126). Neutrophils are among the first inflammatory cells recruited to the site of injury in DKD. Early observational studies indicate that DKD patients exhibit significant neutrophil adhesion (127). Mechanistically, neutrophils release ROS, TNF-α, and neutrophil extracellular trap nets (NETs), which can cause endothelial damage and exacerbate DKD-mediated kidney injury (128, 129). Additionally, studies indicate that an increased neutrophil-to-lymphocyte ratio correlates with more severe proteinuria in outpatient diabetic patients (130, 131).

### 4.2 Intrinsic immune molecules

Monocyte chemotactic protein 1 (MCP-1), or CCL2, is a potent chemotactic factor for monocytes and the first identified human CC chemokine (132). MCP-1 is produced by various cell types, including renal cells, in response to stimuli such as IL-1, IL-4, TNF-α, vascular endothelial growth factor, and INF-γ. Accumulation of AGEs in renal tissues can also induce MCP-1 expression in the context of DKD (133, 134). Earlier studies have shown that urinary MCP-1 levels are significantly elevated in DKD. Moreover, these levels positively correlate with the number of renal interstitial CD68+ macrophages and MCP-1-positive cells. Notably, patients with slower-progressing or less severe forms of DKD exhibit lower urinary MCP-1 levels than those with severe DKD (135). Recent cross-sectional and cohort studies have elucidated the role of MCP-1 in DKD progression. Specifically, MCP-1 serves as a crucial marker for predicting proteinuria development in patients with DM and may also be implicated in the early onset of DKD (136). Additionally, MCP-1 levels significantly correlate with DKD progression (137). Furthermore, the MCP-1 to creatinine ratio is significantly correlated with HbA1c and levels of inflammatory markers (138). Mechanistically, factors such as hypoxia and AGE accumulation induce renal cells to express MCP-1. This protein binds to its corresponding CCR receptor and participates in inflammatory and immune responses by mediating IL-6 production through the NF-κB and activator protein-1 pathways. Additionally, MCP-1 upregulates ICAM-1 expression through the G protein-PKC-Ca^2+^ pathway (139) and enhances endothelial cell E-selectin expression (140). Consequently, it plays a crucial role in the margination, adhesion, quiescence, and migration of rolling monocytes to sites of tissue inflammation. Notably, ICAM-1-deficient mice show inhibited macrophage recruitment, which ameliorates DKD (141). Streptozotocin-induced DKD can be ameliorated through CCR2 knockouts or antagonists (142). Moreover, related drugs, such as CCX140-B, have demonstrated nephroprotective effects in randomized controlled trials (RCT), alongside standard pharmacological agents like ACE inhibitors and ARBs, benefiting patients with DM or DKD (143).

Substantial evidence indicates that serum or urinary levels of inflammatory factors, including IL-1β, IL-6, and TNF-α, are significantly elevated in DKD (144–147). Factors such as high glucose levels, advanced glycation end-products (AGEs), ROS, angiotensin II (Ang II), and a hypoxic microenvironment may trigger the secretion of inflammatory factors by resident or inflammatory cells in renal tissues (148). IL-1 comprises IL-1α and IL-1β, the latter exhibiting stronger pro-inflammatory activity. The synthesis of IL-1 by macrophages occurs in two steps (149). First, TLRs activate the transcription and translation of the IL-1β precursor (pro-IL-1β). Subsequently, nucleotide-binding oligomerization domain-like receptors (NLRs) cleave pro-IL-1β into its active form through a caspase-1-dependent inflammatory vesicle mechanism, thereby exerting pro-inflammatory effects that lead to renal injury and tubulointerstitial fibrosis (150, 151). Inhibiting IL-1β significantly alleviates salt-sensitive hypertension by reducing macrophage polarization toward a pro-inflammatory phenotype (144). The expression of the IL-6 receptor (mIL-6R) is limited to specific cell types, including leukocytes and hepatocytes. Other cells receive IL-6 signals through the soluble IL-6 receptor (sIL-6R). The IL-6-sIL-6R complex in the extracellular fluid binds to gp130 on the cell surface, triggering intracellular signaling through the formation of soluble ligand-receptor complexes, a process termed trans-signaling, or the non-canonical pathway (152). IL-6 facilitates the transition from innate to adaptive immunity via a gp130-STAT3-dependent mechanism, promoting the infiltration of immune cells into renal tissues and remodeling renal architecture (152, 153). Notably, podocytes are the sole glomerular resident cells expressing both mIL-6R and sIL-6R. High glucose stimulation damages podocytes through both canonical and trans-signal transduction pathways, a process reliant on JAK2/STAT3 activation (154, 155). miR-223-3p inhibits IL-6-mediated STAT3 activation, thereby ameliorating injury to human glomerular endothelial cells (156). Additionally, the use of IL-6 inhibitors reduces the incidence of adverse cardiovascular events in patients undergoing renal dialysis (157). TNF-α is primarily synthesized by monocytes and macrophages. Its receptors include the epidermal-type receptor (TNF-R1) and the medullary-type receptor (TNF-R2) (158), with TNF-R1 being the more prevalent receptor. Mechanistically, TNF-α binds to TNF-R1, leading to the upregulation of pro-inflammatory gene transcription through the activation of NF-κB and MAPK signaling pathways. Alternatively, activated TNF-R1 can indirectly stimulate pro-inflammatory gene expression by inducing lytic cell death, releasing DAMPs, and activating neighboring cells via pattern recognition receptors (PRRs) (159). Epidemiological studies indicate that blood levels of TNF-α and its receptors are significantly elevated in T2DM patients with an eGFR of 30-60 mL/min/1.73 m² compared to those with better renal function (160). The TNF-α inhibitor etanercept has been shown to ameliorate aristolochic acid-induced renal fibrosis, which correlates with increased p38 MAPK phosphorylation levels (161). These findings suggest that inhibiting TNF-α may serve as an adjunctive strategy for treating CKD.

IL-10, an anti-inflammatory cytokine secreted by immune cells such as M2-type macrophages and CD4+ Treg cells, binds to IL-10Rα and IL-10Rβ receptors on target cells. This interaction activates intracellular Janus kinase 1 (Jak1) and tyrosine kinase 2 (Tyk2), leading to the phosphorylation of signal transducer and activator of transcription proteins (STAT1, STAT3, and STAT5) (162). This process is critical for IL-10-mediated anti-inflammatory effects and immune regulation. In a mouse model of unilateral ureteral obstruction, IL-10 inhibits inflammatory factor expression and ameliorates renal fibrosis by antagonizing the TGF-β/Smad3 and NF-κB signaling pathways (9). Notably, IL-10 blocks the metabolic reprogramming induced by inflammatory stimuli in macrophages. Furthermore, IL-10 promotes the clearance of dysfunctional mitochondria (163), thereby preventing inflammatory injury associated with mtROS accumulation. TGF-β1 is one of the most abundant isoforms in the TGF-β family, expressed by all renal resident and infiltrating inflammatory cells (164). In the context of DKD, stimuli that promote TGF-β1 expression include Ang II, persistent hyperglycemia, accumulation of AGEs, and ROSs accumulation (10, 165). TGF-β1 is secreted into the ECM as a latent complex comprising the TGF-β latency-associated peptide (LAP) and latent TGF-β binding proteins (LTBP). Inactive TGF-β1 is converted to its active form through cleavage in the presence of integrins, ROS, thrombospondin-1 (TSP-1), and serine proteases. TGF-β1 is uniquely activated by ROS, attributed to a distinct methionine residue at the 253rd amino acid of its LAP, which induces conformational changes in TGF-β binding proteins (166). A high-glucose environment promotes inflammation and fibrosis through ROS production (167, 168). TSP-1 is a multifunctional ECM protein enriched in platelet α-granules and secreted by various cells, including macrophages, fibroblasts, and endothelial cells (169). In DKD, stimuli such as ROS, hyperglycemia, Ang II, hypoxia, and inflammation indirectly activate TGF-β1 by inducing TSP-1 production (170–172). Upon activation, TGF-β1 binds to its receptor, activating downstream signaling through canonical or non-canonical pathways. The canonical pathway is Smad-dependent, with different Smad proteins exhibiting distinct or even opposing roles in the regulation of renal fibrosis (95). Notably, the balance between pro-fibrotic Smad3 and anti-fibrotic Smad7 is crucial to TGF-β1’s role in the progression of renal fibrosis. TGF-β1 signaling contributes to renal fibrosis progression by increasing ECM expression, decreasing degradation, and facilitating dedifferentiation of proximal tubular epithelial and endothelial cells, while also stabilizing collagen cross-linking (10). It promotes inflammation and increases autophagy. Notably, TGF-β1 activates intrinsic renal fibroblast phenotypes, including glomerular mesangial cells, mesangial fibroblasts, and pericytes, driving their proliferation into myofibroblasts that produce collagen in large quantities (173), thereby accelerating renal fibrosis progression.

### 4.3 Intrinsic immune signaling pathways

The JAK/STAT signaling pathway is evolutionarily conserved, comprising ligand-receptor complexes that include Janus kinases (JAKs) and signal transducers and activators of transcription (STATs). The JAK family comprises four members: JAK1, JAK2, JAK3, and TYK2, while the STAT family includes seven members: STAT1, STAT2, STAT3, STAT4, STAT5a, STAT5b, and STAT6 (174). JAK family members include non-receptor tyrosine protein kinase regions. Upon cytokine binding to its receptor, JAK tyrosine kinases are activated, transducing regulatory signals (174). Each STAT member possesses an SH2 domain, primarily responsible for recognizing phosphotyrosine motifs in cytokine receptors (175). This SH2 domain interacts synergistically with activated JAK, facilitating the binding of phosphorylated STAT monomers to form homodimers or heterodimers (176). The JAK-STAT pathway operates fundamentally by cytokines, such as IL-6 (28) and IFN-γ (177), binding to their respective receptors, inducing dimerization and phosphorylating the JAKs. This JAK-mediated phosphorylation recruits and activates STAT, leading to the formation of homo- or heterodimers, which translocate to the nucleus to initiate transcription of target genes (178). In DKD, renal tissues exhibit significantly upregulated expression of JAK2 and STAT3. Specific overexpression of JAK2 in glomerular podocytes of mice leads to manifestations akin to the early stages of human DKD, including increased proteinuria, mesangial thickening, glomerulosclerosis, and accumulation of fibrillar adhesive proteins. Additionally, there is significant thickening of the glomerular basement membrane and a reduced density of podocytes (179). Moreover, high glucose activates the JAK-STAT pathway, inhibiting podocyte autophagy and preventing the effective removal of damaged proteins and organelles. This contributes to cellular stress and death. Conversely, ruxolitinib, a JAK-STAT pathway inhibitor, significantly enhances autophagic flux and autophagy-related gene expression in high-glucose-treated podocytes (180), alleviating renal injury. A phase II clinical trial evaluating baricitinib for DKD treatment (181) found that, after 24 weeks, baricitinib significantly reduced the morning UACR and inflammatory markers, including urinary CCL2, serum TNF-R1, TNF-R2, and ICAM levels, compared to healthy controls. Consequently, baricitinib reduced albuminuria and ameliorated renal injury in patients with DKD. Suppressors of cytokine signaling (SOCS) function as endogenous inhibitors of the JAK-STAT pathway. Down-regulation of SOCS can activate the JAK1-STAT1 pathway, promoting the polarization of renal macrophages toward the M1 phenotype (182) and exacerbating inflammatory injury. Conversely, up-regulating SOCS to inhibit the JAK-STAT pathway has been proposed as a potential therapeutic strategy for DKD (183).

Toll-like receptors (TLRs) are a group of receptors that recognize molecular patterns in innate immunity. They contribute to aseptic inflammation by binding to endogenous ligands, such as damage-associated molecular patterns (DAMPs), as well as to pathogenic infections through exogenous receptors. TLR4 is currently the most extensively studied and significant member of the TLR family (184). TLR4 is classified as a type I transmembrane receptor consisting of three regions: extracellular, intracellular, and cytoplasmic. The cytoplasmic region is also known as the Toll/interleukin-1 receptor (TIR) domain. In diabetic kidney disease (DKD), conditions such as hyperglycemia, hypoxia, and lipid metabolism disorders upregulate levels of TLR4 ligands, including HMGB1, biglycan, and S100 calcium-binding protein (S100A8) (185–187). TLR4 binding to ligand molecules induces conformational and charge distribution changes that activate the cytoplasmic TIR region, subsequently initiating downstream pathways via either the myeloid differentiation factor 88 (MyD88)-dependent or MyD88-independent pathways (188). The MyD88-dependent mechanism involves two adapter proteins binding to the intracytoplasmic portion of TLR4: MyD88 and the TIR-containing adapter protein (TIRAP). This interaction recruits and activates IL-1 receptor-associated kinase 4 (IRAK4), leading to the activation of IRAK1 by phosphorylated IRAK4 (REF (189)). IRAK1 further recruits TNF receptor-associated factor 6 (TRAF6), resulting in the activation of downstream inhibitory kappa B kinase (IKK) and MAPK pathways. IKK, a complex composed of IKKα and IKKβ, regulates the NF-κB pathway and is also referred to as the NF-κB essential modulator (NEMO). NF-κB comprises five members: p50, p52, p65 (REL-A), REL-B, and c-REL, with the p65/p50 heterodimer being the most prevalent and significant. Under normal conditions, IκB binds to NF-κB and sequesters it in the cytoplasm (190). Upon TLR4 activation, IKK phosphorylates IκB, promoting its proteasomal degradation. This allows NF-κB to translocate to the nucleus and initiate the transcription of inflammation-related genes (191). Additionally, activated MAPK exerts pro-inflammatory activity by phosphorylating the transcription factor complex activator protein-1. This complex translocates to the nucleus and further induces the expression of inflammatory genes, including TNF, IL-6, IL-1β, and various chemotactic molecules. The MyD88-independent pathway involves TIR-containing adaptor inducing interferon-β (TRIF) and TRIF-related adaptor molecule (TRAM) (192). TLR4 activation signals activate TRAF3 via TRIF and TRAM, ultimately inducing IFN-β expression through associated pathways. Notably, renal TLR4 expression is significantly increased in patients with DKD compared to healthy controls. Mechanistically, high glucose activates TLR4 and upregulates TLR4 protein, IL-6, and MCP-1 expression via the PKC/IκB/NF-κB pathway, facilitating renal interstitial macrophage infiltration and inflammatory responses (191, 193). Conversely, inhibiting TLR4 significantly reduces renal interstitial fibrosis (191).

The NLRP3 inflammasome is the most extensively studied inflammasome to date. NLRP3 is a protein characterized by a nucleotide-binding oligomerization domain (NOD), a leucine-rich repeat (LRR), and a pyrin domain. It is classified as a cytoplasmic PRR. The NLRP3 inflammasome, in contrast, comprises one sensor (NLRP3), one adaptor protein (ASC), and an effector molecule (caspase-1) (194, 195). The activation state of the inflammasome depends on a complex regulatory mechanism characterized by two phases: initiation and activation. In the initiation phase, TLR ligands such as TNF-α, IL-1β, and HMGB1, which are upregulated in DKD, activate TLRs, subsequently activating IKK through specific signaling pathways. This activation facilitates the translocation of NF-κB into the nucleus, enhancing the expression of pro-inflammatory precursor molecules, including pro-IL-1β, pro-IL-18, and NLRP3 (185, 187, 196). Additionally, this phase can also induce self-repression of NLRP3 through post-translational modifications (195). During the subsequent activation phase, the expression and activity of NLRP3 are upregulated to prepare for full activation. However, most tissue cells cannot activate NLRP3 inflammasomes and instead constitutively express IL-1β due to weak initiation signals (197). A consistent model for the NLRP3 activation phase remains elusive. During DKD, sustained high glucose levels, lipid metabolism disorders, and tissue hypoxia are potential triggers (5). Mechanisms of activation may involve potassium or chloride ion efflux, calcium ion influx, lysosomal and mitochondrial dysfunction, and endoplasmic reticulum stress (195, 198). Following NLRP3 inflammasome activation, caspase-1 cleaves pro-IL-1β and pro-IL-18 into their mature forms, releasing them into the extracellular space and exacerbating inflammatory damage. Knockout of TLR4 improves high glucose-induced podocyte apoptosis and enhances cell viability via the NLRP3/ASC/caspase-1 signaling pathway (199). Shahzad et al. discovered that mice with podocyte-specific overexpression of NLRP3 exhibited increased proteinuria, mesangial proliferation, and basement membrane thickening. In contrast, mice with podocyte-specific knockout of NLRP3 or caspase-1 displayed renal protection. Additional cellular experiments indicate that podocyte-specific NLRP3 inflammasome activation may promote aseptic inflammation and glomerular injury in DKD kidney tissue (200). Furthermore, a high-glucose environment leads to ROS accumulation, which induces NLRP3 inflammasome activation, activates caspase-1, mediates IL-1β synthesis and secretion, and induces HK-2 cell apoptosis (148). Inhibiting mitochondrial ROS production by knocking out CD36 can suppress inflammasome activation and improve tubulointerstitial inflammation and apoptosis of renal tubular epithelial cells in db/db mice.

The canonical activation pathway of TGF-β1 primarily involves Smad proteins 2, 3, 4, and 7. Activated TGF-β1 binds to the type II membrane receptor (TβRII), subsequently recruiting and phosphorylating the type I TGF-β receptor (TβRI). This activated TGF-β1-TβRII-TβRI complex phosphorylates Smad2 and Smad3 while binding to Smad4 to form Smad complexes. These Smad complexes translocate to the nucleus, where they bind to Smad-binding elements (SBEs) to regulate gene transcription (10), including the synthesis of collagen, fibronectin, and α-smooth muscle actin (α-SMA). While Smad2, Smad3, and Smad4 proteins exert regulatory effects by forming complexes, they can have distinct or even opposing effects. Notably, Smad3 can directly bind to the promoter region of SBEs to enhance transcription, whereas Smad2 and Smad4 lack DNA-binding domains and instead regulate the transcription of Smad3-targeted genes (201, 202). Among Smad proteins, Smad7 can compete with Smad2 and Smad3 for binding and activation of TβRI, providing negative feedback regulation of the TGF-β/Smad classical signaling pathway (203). Due to ubiquitin-mediated degradation, the expression of Smad7 in the renal tissue of STZ-induced DKD animals is significantly reduced. The latent TGF-β1 complex can ameliorate kidney injury by inhibiting Arkadia-mediated Smad7 ubiquitin degradation (204), thereby upregulating Smad7 expression.

## 5 Interaction between intrinsic immunity and ferroptosis in DKD

### 5.1 Ferroptosis and intrinsic immune cells

**Figure 3:**
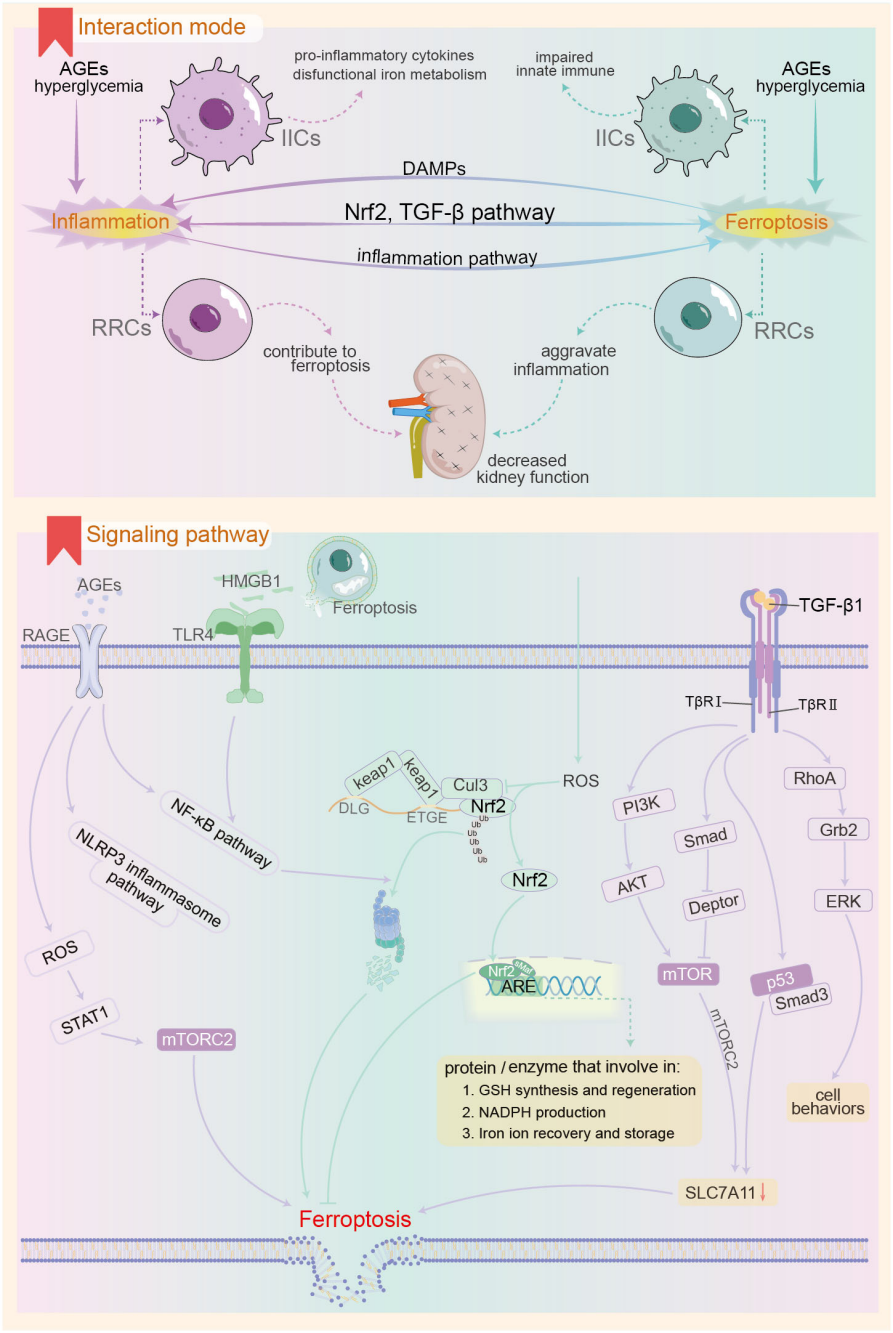
Possible mechanisms of interaction between ferroptosis and innate immune inflammatory response in the context of DKD. The upper panel demonstrates the pattern of ferroptosis-inflammation interaction in the context of DKD, and the lower panel shows the important pathways involved in the interaction between the both. AGEs, advanced glycation end products; RAGE, receptor for AGEs; IIC, innate immune cells; RRCs, renal resident cells; DAMPs, Damage-Associated Molecular Patterns; Nrf2, Nuclear factor erythroid 2-related factor 2; mTOR, mammalian target of rapamycin; keap1, kelch-like ECH-associated protein-1; Cul3, Cullin-3; PI3K, Phosphoinositide 3-kinase; RhoA, Ras homolog gene family member A; Grb2, growth-factor receptor-bound protein-2; ERK, extracellular signal-regulated kinase.

Macrophages influence renal cell ferroptosis by maintaining iron ion metabolism homeostasis in both systemic and local renal environments and secreting immune molecules (**Figure 3**). Phosphatidylserine expression on aging red blood cells (RBCs) serves as an “eat me” signal (205), promoting macrophage phagocytosis in the spleen. Macrophages break down red blood cells and release iron ions back into circulation. In diabetes mellitus, chronic inflammation may stimulate macrophage clearance of RBCs. Concurrently, decreased hematopoietic function of the bone marrow and reduced charge selectivity of the glomerular filtration membrane lead to excess iron ions in renal tubular fluid and tissue. Furthermore, a lack of the labile iron pool (LIP) in renal macrophages (206), along with the outward migration of iron ions and abnormal iron metabolism in systemic and local macrophages, promotes renal iron overload and ferroptosis; circulating iron, however, remains deficient due to loss. Pro-inflammatory cytokines from M1 macrophages, including IL-1β, IL-6, and TNF-α, regulate the synthesis of iron homeostasis-associated proteins and play a role in ferroptosis regulation (207). Macrophages are involved in clearing various forms of dead cells, where SAPE-OOH molecules on the surface of ferroptosis cells act as “eat me” signals, promoting macrophage phagocytosis (208). The distinct clearance characteristics of macrophages can provide a basis for differentiating among various forms of cell death (209). Ferroptosis affects macrophages by facilitating the binding of DAMPs released from dissolved and ruptured cells to TLRs. This binding leads to the release of inflammatory factors such as IL-1β and TNF, promoting macrophage polarization toward M1 pro-inflammatory phenotypes. Additionally, macrophages themselves can undergo ferroptosis, which suppresses innate immunity (210). Inhibiting macrophage ferroptosis can alleviate macrophage-mediated renal tubular epithelial-to-mesenchymal transition (211) and downregulate fibrosis-related gene expression.

### 5.2 Ferroptosis and intrinsic immune molecules

DKD mice with IL-1 receptor antagonist knockout exhibited severe renal dysfunction and anemia compared to wild-type DKD. This was accompanied by downregulation of renal hypoxia-inducible factor-2 and decreased expression of the bone marrow erythropoietin receptor and TFR (212). This downregulation can lead to intracellular iron accumulation and promote oxidative free radical production via the Fenton reaction. Administering the IL-1β monoclonal antibody, P2D7KK, improves systemic inflammation, renal function, and corrects anemia. IL-6 is a crucial immune molecule that regulates systemic iron metabolism during inflammatory responses. It binds to mIL-6R on liver cells, activating hepcidin expression via the JAK/STAT3 pathway (28). Elevated hepcidin in the renal circulation promotes the internalization and degradation of FPN1 from the membrane (213), potentially resulting in intracellular iron overload and ferroptosis in renal cells. In the context of chronic inflammation, insufficient expression of the anti-inflammatory cytokine IL-10 leads to downregulation of DDIT4, an mTOR inhibitory factor, in macrophages. This upregulates mTOR activity (163), which may inhibit xCT subunit activity, diminish cell resistance to lipid peroxides, and promote ferroptosis. Additionally, insufficient IL-10 expression may coincide with reduced mitochondrial autophagy, leading to decreased membrane potential and significant accumulation of ROS in mitochondria (214). This promotes abnormal NLRP3 inflammasome activation, resulting in IL-1β production and subsequent inflammatory damage.

High mobility group box 1 (HMGB1) is a nuclear protein that binds DNA and functions as a transcription cofactor. Under normal circumstances, HMGB1 resides exclusively in the nucleus and participates in various biological processes, including DNA repair and gene transcription (215). Highly conserved through evolution, HMGB1 is released by monocytes/macrophages undergoing lytic cell death or activation, subsequently triggering innate and inflammatory immune responses. HMGB1 plays a crucial role in the body’s endogenous resistance to adverse stimuli (216). An observational study indicated that serum and urine HMGB1 levels in DKD patients were significantly elevated compared to those in diabetic non-nephrotic individuals or healthy controls (89, 217). Mechanistic studies have shown that high glucose levels can upregulate HMGB1 expression in renal mesangial and tubular epithelial cells, leading to its abnormal localization in the cytoplasm and extracellular fluid (218). Furthermore, ferroptosis, a form of regulated cell death (219), occurs when the cell membrane ruptures due to lipid peroxide accumulation. Among the released cellular contents, HMGB1 is present (220). HMGB1 is recognized as a DAMP, with receptors such as RAGE, TLR-2, -4, and CXCR4 (215, 221). By binding to various receptors, HMGB1 elicits diverse effects, including chemotactic recruitment of inflammatory cells, upregulation of inflammatory levels, and promotion of fibrotic proliferation (215, 216, 222). Following receptor binding, HMGB1 upregulates pro-inflammatory gene expression by activating the NF-κB or NLRP3 inflammasome pathways, contributing to renal inflammatory damage and epithelial-mesenchymal transition (223). Inhibition of HMGB1 action using competitive antagonists (224) can effectively delay DKD progression. Beyond inducing innate immune activation, HMGB1 regulates high glucose-induced ferroptosis in glomerular mesangial cells. Mechanistically, HMGB1 activates the TLR4/NF-κB signaling pathway, leading to increased Nrf2 ubiquitination degradation, evidenced by downregulation of GPx4 and upregulation of ASCL4 expression, along with a decreased capacity for cellular iron ion storage (20, 225). Consequently, HMGB1 promotes ferroptosis in mesangial cells by disrupting intracellular iron homeostasis, impairing ferroptosis-related antioxidant systems, and increasing the synthesis of polyunsaturated fatty acid phospholipid peroxides. Montelukast targets the HMGB1/TLR4/NF-κB and inflammasome pathways to inhibit STZ-induced renal inflammation and fibrosis progression in DKD rats (226). Concurrently, montelukast can reduce levels of the ferroptosis marker MDA, indicating a significant improvement in the redox system.

### 5.3 Ferroptosis and intrinsic immune signaling pathways

Advanced glycation end products (AGEs) are formed through non-enzymatic reactions between reducing sugars, their metabolites, and proteins or amino acids. Major AGEs include methylglyoxal-derived hydroimidazolone (MG-H), Nϵ-(carboxymethyl)lysine (CML) derived from Nϵ-fructosyl lysine, and crosslinked glucosepane (227). Under normal conditions, dietary intake is the primary source of AGEs (228). In DKD, prolonged hyperglycemia accelerates protein glycosylation. Concurrently, renal function decline reduces the excretion of AGEs and their precursors, such as methylglyoxal, resulting in significant AGE accumulation—a notable feature in DKD patients (229). As post-translational modifications, AGEs persist in the body and may damage various tissues, particularly the kidneys (230). AGEs are closely linked to CKD and DKD (231). The receptors for AGEs include RAGE (receptor for AGE) and AGER (AGE receptor), with the former being extensively studied (232). Following AGE-RAGE activation, immune cell infiltration occurs through the upregulation of MCP-1 and ICAM-1 on endothelial cells in renal tissue (233). Additionally, it can directly induce oxidative stress in kidney cells by activating the NF-κB and NLRP3 inflammasome pathways (234). This activation enhances the release of inflammatory factors such as IL-1β, IL-18, and TNF-α, while also indirectly causing kidney damage. It achieves this by increasing macrophage cytoplasmic STAT1 phosphorylation and promoting the expression of inflammatory factors like IL-6 and TNF-α via the RAGE/ROS/TLR-4/STAT1 signaling pathway (235). Consequently, some researchers identify RAGE as a pattern recognition receptor (236). Additionally, AGEs downregulate endothelial nitric oxide synthase (eNOS) in renal cells, resulting in diminished NO synthesis and impaired endothelial relaxation. Conversely, AGEs/RAGE activation upregulates iNOS, thereby promoting inflammation. Studies indicate that AGEs activate the nuclear factor STAT5, upregulate the expression of cardiomyocyte-related transcription factor A, and damage glomerular mesangial cells (237). Furthermore, AGEs downregulate zonula occludin-1 expression via the PI3K/Akt signaling pathway, damaging the podocyte filtration barrier (238). This increases permeability and exacerbates proteinuria. Lowering serum AGEs levels through dietary modifications or AGE inhibitors (239), as well as utilizing RAGE antagonists (228, 234), significantly improves macrophage infiltration, reduces inflammation, and alleviates kidney damage. Regarding physiological parameters, the accumulation of AGE-related protein content can lead to renal hemodynamic disorders and promote the progression of DKD (240). Additionally, studies on the effects of high glucose or AGEs on glomerular mesangial cells and human tubular epithelial cells observed activation of inflammatory pathways, including NF-κB. Notably, MDA levels in the experimental group increased significantly, while SOD and GPx levels decreased (241). This suggests that AGEs heighten the sensitivity of renal cells to ferroptosis. Existing evidence suggests that, in the context of diabetes, accumulated AGEs promote complications by inducing ferroptosis, including diabetic cardiomyopathy and osteoporosis (242, 243). Further research is warranted to elucidate the mechanisms by which AGEs induce ferroptosis in renal cells and to determine whether this process significantly contributes to the pathogenesis of DKD.

Nrf2, a key member of the basic region leucine zipper (bZIP) family, plays a crucial role in combating intracellular oxidative stress (244). It belongs to the CNC (cap ‘n’ collar) subfamily, comprising seven functional regions known as Neh1-7. The Neh1 region, highly conserved, includes the CNC-bZIP domain involved in binding target genes and interacting with Nrf2 co-factors, such as the small musculoaponeurotic fibrosarcoma (sMaf) protein. The Neh2 region features two motifs, DLG and ETGE, that bind to Keap1, with ETGE exhibiting a stronger affinity (245). Neh3, Neh4, and Neh5 work synergistically to regulate the expression of Nrf2 target genes. Neh6 negatively regulates Nrf2 independent of Keap1, while Neh7 interacts with retinoid X receptor alpha, thereby inhibiting Nrf2 activity (244, 246). Nrf2 activation differs from conventional phosphorylation by protein kinases. Several hypotheses have emerged to elucidate the mechanisms of electron affinity activation of Nrf2 during oxidative stress, including the “hinge and latch” hypothesis (247) and the “conformational cycling model” (248). For example, under normal conditions, Keap1 homodimers bind to the ETGE and DLG motifs in the Neh2 region of Nrf2. ETGE is tightly bound to Keap1 (hinge), while DLG is loosely associated (latch). Thus, two Keap1 proteins and one Nrf2 form a complex. Mediated by Cullin-3 (Cul3), an E3 ligase, Nrf2 undergoes ubiquitination and subsequent degradation by the proteasome. Under oxidative stress, the electrophilic ligand disrupts the binding between DLG and Keap1. Although Nrf2 remains associated with Keap1 through ETGE, it cannot undergo ubiquitination, thus blocking its degradation pathway. Consequently, newly synthesized Nrf2 can translocate to the nucleus, where it interacts with the sMaf protein and other bZIP transcription factors to form a transcriptional activation complex. This complex subsequently binds to the ARE sequence in the promoter region of target genes, thereby upregulating their expression (247, 249, 250). Nrf2 regulates numerous target genes, all of which contain cis-acting enhancer sequences (5’-TGACnnGC-3’), a characteristic of ARE. The products of Nrf2 target genes can be categorized into three broad groups (250). First, proteins involved in the synthesis and regeneration of GSH (251), including GCL complex modification subunits, catalytic subunits, GSH reductase, xCT, GPx, and glutathione S-transferase (GST) (45). Second, enzymes that facilitate oxidation-reduction processes, such as thioredoxin and peroxide-reducing protein 1, are also regulated by Nrf2. Third, proteins involved in iron ion recovery and storage, including heme oxygenase-1 (HO-1) and ferritin heavy and light chains (250, 252). Thus, Nrf2 is closely tied to iron homeostasis and the clearance of lipid peroxides. Oxidative stress, inflammation, and innate immunity frequently interplay (250), making the Nrf2 pathway a crucial bridge linking ferroptosis and inflammatory stress. Persistent chronic inflammation in renal tissue is critical to the onset and progression of DKD. Observational studies indicate that Nrf2 expression is suppressed in DKD patients (88). In an Akita mouse model, Nrf2 knockout mice exhibited greater glomerular interstitial dissolution, mesangial proliferation, and increased tubular hyaline change, alongside heightened macrophage infiltration compared to control mice. In contrast, expression levels of GSH and its synthesis-related genes were markedly downregulated. Conversely, mice expressing higher levels of Nrf2 demonstrated significant renal tubular injury improvement, often lacking any tubular hyaline cast (253). Upregulation of Nrf2 through fenofibrate treatment inhibited ferroptosis in diabetic kidney cells, subsequently delaying DKD progression (254). These findings suggest that Nrf2 downregulation is a key contributor to oxidative stress and ferroptosis in renal tissue during DKD. Future research is essential to elucidate Nrf2 level changes in the renal tissue of DKD patients at various stages and to assess the distinct effects and sensitivity of Nrf2 pathways to oxidative stress across different cell types.

The TGF-β1/Smad signaling pathway interacts with several other signaling pathways. The mitogen-activated protein kinase (MAPK) family includes three major kinases: p38 MAPK, c-Jun N-terminal kinase (JNK), and extracellular-signal-regulated kinase (ERK). These kinases are activated by Ang II, AGEs, and pro-inflammatory cytokines, leading to the upregulation of TGF-β1 in various renal cell types, including mesangial and tubular epithelial cells. Once activated, TβRI functions as a tyrosine receptor kinase through phosphorylation, thereby activating the RhoA/Grb2/ERK signaling pathway (255) as well as p38 MAPK and JNK via the TNF receptor-associated factor 6 and TGF-β-activating enzyme-1/M3K7 pathways (256, 257). The mammalian target of rapamycin (mTOR) is a serine/threonine kinase comprising two distinct protein complexes: mTOR complex 1 (mTORC1) and mTOR complex 2 (mTORC2). These complexes independently activate downstream signaling pathways (258). Inhibition of mTORC1 activity by rapamycin promotes longevity, while inhibition of mTORC2 activity induces insulin resistance and disrupts glucose homeostasis (259). TGF-β1 activates the mTOR signaling pathway via the canonical Smad-dependent mechanism by inhibiting Deptor expression, a repressor of both TORC1 and TORC2. Additionally, it stimulates mTOR through the non-canonical PI3K/AKT pathway, promoting glomerular mesangial cell proliferation (95). Conversely, mTORC2 phosphorylates the xCT subunit, leading to its inhibition, which contributes to ferroptosis (50). The tumor suppressor p53 regulates cell differentiation, proliferation, and apoptosis. TGF-β1 induces phosphorylation and acetylation of p53 in renal tubular epithelial cells, forming the p53/Smad3 complex and promoting the expression of pro-fibrotic factors, a phenomenon observed in both renal tubular epithelial cells and fibroblasts (260–262). Evidence indicates a positive feedback loop among TGF-β1, miRNA-192, and p53 that accelerates the progression of diabetic kidney disease (DKD) (263). Meanwhile, p53 activation negatively regulates xCT, thereby increasing sensitivity to ferroptosis (47). In conclusion, TGF-β1 in DKD regulates inflammatory and fibrotic processes through the Smad pathway while also interacting with the p53 and mTOR pathways to modulate ferroptosis.

## 6 Drugs targeting ferroptosis and intrinsic immunity for the treatment of DKD

### 6.1 Sodium-glucose cotransporter protein-2 inhibitor

Sodium-glucose cotransporters (SGLTs) are transmembrane proteins crucial for transporting sodium ions and glucose (264). The two predominant isoforms, SGLT-1 and SGLT-2, have SGLT-2 highly expressed on the luminal surface of proximal tubular cells, responsible for approximately 90% of glucose reabsorption in glomerular filtrate. This process recycles absorbed sodium ions back into circulation through the basolateral Na^+^/K^+^ ATPase (265). This active transport process consumes energy, contributing to a hypoxic microenvironment in the kidney due to elevated glucose levels in glomerular ultrafiltrate, coupled with increased SGLT2 load and energy demand in DM. When glucose reabsorption transporters like SGLT2 fail to adequately handle the excess tubular fluid glucose, it results in glucose appearing in urine. Mechanistically, SGLT2i reduce glucose reabsorption by inhibiting SGLT2, effectively lowering blood glucose levels. Nonetheless, large clinical trials on SGLT2i continue to reveal new insights. Early studies investigating the effects of SGLT2i on cardiovascular disease outcomes in T2DM indicated that agents such as empagliflozin (266), dapagliflozin (267), and canagliflozin (268) significantly reduced the incidence of major adverse cardiovascular events, including cardiovascular death and myocardial infarction. This protective effect was particularly pronounced among patients with T2DM and poorer eGFR. Moreover, one study indicated that the renal composite outcome—including greater than 40% reduction in eGFR, renal replacement therapy, and death due to renal disease—was significantly improved in the trial group. Additionally, SGLT2i-related clinical trials focusing on adverse renal outcomes have demonstrated that, regardless of heart failure history (269), the SGLT2i group significantly delayed eGFR decline, improved proteinuria, and reduced the risks of ESRD, renal failure, and death due to renal disease compared to the placebo group (270–273). Consequently, the 2022 authoritative guidelines recommend SGLT2i as first-line agents for patients with diabetic CKD (274, 275). While the role of SGLT2 analogues in enhancing renal function in patients with DKD has been clinically validated and widely recognized, the specific mechanisms underlying their multi-organ benefits remain under investigation.

Research on the renal benefits of SGLT2i indicates that, beyond significantly lowering blood glucose levels and improving renal hemodynamics, the amelioration of inflammatory damage is a crucial contributing factor (276). Prospective cohorts (277–279) and small clinical trials (25) have demonstrated that patients receiving SGLT2i treatment exhibited notable reductions in serum inflammatory markers, such as TNF-α, TNFR1, IL-1, and IL-6, compared to control groups. A recent meta-analysis encompassing 30 studies examined the effects of SGLT2i drugs on common inflammatory markers in preclinical settings (280). The results revealed that the administration of SGLT2i agents, particularly dapagliflozin and empagliflozin, significantly lowered levels of MCP-1, IL-6, TNF-α, and C-reactive protein in rodent models. In patients with DKD, those treated with dapagliflozin showed a marked reduction in the urinary MCP-1/creatinine (Cr) ratio and IL-6/Cr ratio compared to placebo controls. This suggests that dapagliflozin alleviates renal inflammatory cell infiltration and inflammatory stress (281). The mechanisms through which SGLT2i mitigates renal inflammatory injury involve enhancing podocyte autophagy, promoting M2 cell polarization, and inhibiting the HMGB1-TLR4 inflammatory pathway (282–285). Moreover, research has explored the effect of SGLT2i on ferroptosis. Cross-sectional studies have revealed that SLC7A11 and GPx4 expression is significantly downregulated in DKD kidney biopsy tissues compared to those from non-diabetic nephropathy patients (286). In a prospective cohort study (278), diabetic patients on empagliflozin exhibited an increase in the serum anti-inflammatory molecule IL-10 and a decrease in blood leukocyte peroxides by week 24. GSH levels and GSH reductase activity also rose, indicating that empagliflozin may enhance the antioxidant capacity of diabetic patients (278) and potentially bolster the body’s resistance to ferroptosis. Animal and cellular experiments have demonstrated that SGLT2i drugs can ameliorate renal tubular ferroptosis in DKD through various pathways. For instance, canagliflozin activates the AMPK energy-regulated pathway (286), stimulating Nrf2 activity and subsequently upregulating GPx4, FTH, and SLC7A11 expression. Dapagliflozin inhibits the ubiquitylation-degradation of SLC7A11 (26), thereby stabilizing this protein. What’s more, canagliflozin improves fatty acid oxidation via the FOXA1-CPT1A axis (287) and mitigates renal tubular ferroptosis in DKD. Notably, recent evidence indicates that dapagliflozin downregulates the overactivated HIF1α/HO-1 axis in DKD, alleviating ferroptosis in renal tissues and mitigating disease progression (288).

### 6.2 Inhibition of the JAK-STAT pathway

Baricitinib, an oral small-molecule inhibitor of the JAK family of protein tyrosine kinases, has shown clear clinical efficacy in treating chronic inflammatory diseases, such as rheumatoid arthritis, via selective inhibition of JAK1 and JAK2 (289, 290). A clinical phase II double-blind, dose-ranging study explored the effects of baricitinib on proteinuria in T2DM patients at high risk of DKD progression. In total, 129 DKD participants, with an average age of 63 years, were enrolled, exhibiting baseline HbA1c and eGFR levels of 7.3 ± 1% and 45.0 ± 12.1 ml/min/1.73 m², respectively (181). After 24 weeks, the baricitinib group demonstrated a reduction in morning urine UACR of up to 41% compared to the placebo control group and showed lower albuminuria. Notably, the effects on UACR and albuminuria were maintained for four weeks post-discontinuation of the drug, suggesting that baricitinib may be nephroprotective (181, 291). Furthermore, the baricitinib group displayed significantly reduced inflammatory markers, including lower serum ICAM and urinary CCL2, indicative of improved renal innate immune cell infiltration (141). Additionally, lower serum TNF-R1 and TNF-R2 levels suggested a reduction in both renal tissue and systemic inflammation (161). Mechanistically, JAK inhibitors downregulate the activation of the JAK-STAT pathway in podocytes in response to high-glucose stimulation, leading to decreased pro-inflammatory gene expression and increased autophagy (180). Baricitinib specifically inhibits the JAK/STAT3 pathway (292) and may stabilize the labile iron pool by inhibiting iron-modulating hormone expression and upregulating FPN1 on the membrane, thereby counteracting ferroptosis. However, renal function indicators such as eGFR were not significantly altered in the baricitinib group compared to the placebo group (181). Consequently, further studies are warranted to assess the effects of JAK inhibitors on stricter endpoints, including eGFR, ESRD, and nephrogenic death. Baicalin, a flavonoid glycoside derived from Scutellaria baicalensis Georgi, is believed to exert multiple therapeutic effects, primarily due to its anti-inflammatory properties. Baicalin has been shown to ameliorate high glucose-induced kidney injury by inhibiting pro-inflammatory gene expression via the NF-κB and STAT3 pathways (293). Conversely, observational studies indicated that DKD patients receiving baicalein had significantly higher serum levels of superoxide dismutase (SOD) and GPx compared to control DKD patients on standard medication, alongside significantly lower NF-κB levels after six months of treatment (294). This suggests that baicalein enhances antioxidant levels in renal tissues and the systemic circulation, thereby inhibiting ferroptosis. Further investigation is necessary to determine whether baicalein can mitigate renal injury in DKD by inhibiting ferroptosis in renal cells.

### 6.3 Activation of Nrf2 pathway

Bardoxolone methyl (BM), the most extensively studied Nrf2 activator, belongs to a class of synthetic triterpenoid antioxidant modulators. Its capacity to effectively activate the Nrf2 pathway suggests that BM may regulate intracellular ROS levels and mitigate inflammatory damage (295). Additionally, BM inhibits the activation of the NF-κB and JAK/STAT pathways by down-regulating IKKβ and JAK1 protein levels. Early preclinical studies indicate that BM can induce differentiation, inhibit proliferation, and promote apoptosis in various cancer cell lines (296). In a Phase 1 clinical trial designed to identify the appropriate dose of BM (297), patients in the BM group exhibited a notable increase in serum NQO1 levels—one of the downstream target genes of Nrf2—reflecting successful activation of the Nrf2 pathway. Although the anticipated tumor response was suppressed, the study observed a significant increase in eGFR among participants receiving BM, highlighting its potential benefits for CKD. A subsequent Phase 2 double-blind RCT—the BEAM trial (NCT00811889) (298)—investigated the long-term effects of BM in individuals with CKD. It reported a significant improvement in eGFR in the BM group compared to the control, with mild muscle spasms being the most common adverse effect. Gastrointestinal issues followed closely. Notably, atrial fibrosis and renal failure events occurred exclusively in the BM group, even though the authors suggested that serious adverse reactions did not significantly differ between groups (298). The success of the BEAM trial laid the groundwork for a Phase 3 double-blind RCT aimed at assessing whether BM could reduce the risk of progression to ESRD or cardiovascular mortality among patients with stage 4 DKD (the BEACON trial, NCT01351675) (299). Although the trial enrolled 2,185 participants, it was prematurely terminated due to a higher incidence of cardiovascular deaths in the BM group (27) compared to the placebo group (19). Data indicated that BM significantly increased the risk of hospital admissions or death from heart failure (HR = 1.83, 95% CI = 1.32 - 2.55) (299). However, analyzing the role of BM in detail was complicated by the lack of baseline cardiac function metrics, such as echocardiography. The specific mechanisms through which BM may induce myocardial injury remain unclear. In patients with advanced DKD, factors such as high glucose levels, urinary toxin accumulation, and altered hemodynamics may heighten the risk of adverse events, including heart failure and sudden cardiac death. Consequently, the key pathways regulated by BM may render cardiac tissue more susceptible to these detrimental conditions.

The exploration of BM continues (300), with in-depth analyses of adverse side effects from the BEAM and BEACON trials. Notably, in BEACON, both eGFR and UACR were significantly higher in the BM group, which also observed fewer patients progressing to ESRD (43) compared to the placebo group (51). This suggests the presence of a nephroprotective effect of BM, although the impact on cardiac function in subjects was tightly constrained. Consequently, a phase 2 double-blind RCT, known as the TSUBAK trial, was conducted to evaluate whether BM could mitigate fluid retention risk and enhance eGFR in patients not at risk of heart failure (301). This trial implemented stringent exclusion criteria regarding cardiac function, including excluding patients with a baseline BNP >200 pg/ml or a history of severe cardiovascular disease. The results indicated no serious adverse events and a significant increase in eGFR within the BM group. These findings are promising for further clinical trials assessing the long-term safety and efficacy of BM in DKD under strict cardiac function limitations. For instance, the AYAME trial (NCT03550443) completed in July 2023, as referenced in the Clinical Trials Registry (https://www.clinicaltrials.gov/), is currently collating its results and is expected to publish findings soon (302).

### 6.4 Inhibition of AGEs/RAGE pathway

The accumulation of AGEs is a prominent characteristic of diabetes and its complications, particularly DKD. This phenomenon arises from the reduced renal excretion of AGEs and their precursors, leading to significant accumulation that exacerbates renal inflammatory injury (234). High serum levels of AGEs and soluble receptor for advanced glycation end-products have been associated with an increased risk of renal disease progression in patients with T2DM, with hazard ratios (HRs) of 1.21 and 1.20, respectively (303). Importantly, reducing AGEs or inhibiting their pathways can ameliorate renal function (228, 239). A clinical trial explored the potential of sevelamer carbonate to mitigate the AGEs-induced progression of diabetic CKD through an open-label, intention-to-treat crossover study design. The results indicated that sevelamer carbonate effectively inhibited intestinal absorption of AGEs by binding to them, without altering baseline medication, caloric intake, or AGEs consumption (241, 242). Consequently, the intervention group exhibited a significant reduction in AGEs and their precursors, along with improved markers of inflammation and oxidative stress (304). These findings suggest that sevelamer carbonate may play a role in slowing the progression of DKD.

Starowicz et al. screened 14 biological extracts commonly used in European cuisine for active ingredients that effectively inhibit AGEs. They found that cinnamon, cloves, and pimento exhibited high anti-glycosylation activity (305), offering beneficial alternatives for daily diets aimed at reducing AGEs. Catalpol, an extract from Rehmannia glutinosa, regulates nitric oxide production and ameliorates AGE-induced impairment of renal tubular endothelial function. This occurs through activation of the PI3K/Akt/eNOS signaling pathway and inhibition of the NF-κB/iNOS pathway (306). 4′-Methoxyresveratrol inhibits RAGE-mediated activation of the NF-κB and NLRP3 inflammasome pathways, exerting anti-inflammatory effects and countering AGE-induced inflammatory responses in macrophages (307). Cinnamaldehyde, the primary active constituent of Cinnamomum osmophloeum, protects renal tubular cells from AGE-induced damage by activating the NO pathway. This occurs through inhibition of JAK2-STAT2/STAT3 activation and upregulation of SCOS-3 protein levels (308). The loss of klotho is closely associated with CKD. Osthole, an anticancer and anti-inflammatory drug, ameliorates AGE-induced renal tubular cell hypertrophy by inducing upregulation of klotho and both SCOS1 and SCOS3 expression (309). Additionally, the biosynthetic drug gliclazide ameliorates high glucose or AGE-induced renal cell injury by inhibiting the RAGE-NADPH oxidase-NF-κB pathway (241).

## 7 Conclusion and perspective

Diabetic kidney disease is a prevalent complication of diabetes globally, significantly impacting patients’ health and quality of life. Factors such as persistent hyperglycemia and the accumulation of AGEs lead to the infiltration of inflammatory cells, particularly innate immune cells, triggering a subsequent inflammatory response in renal tissues. These processes are key mechanisms underlying inflammatory stress and the progression of fibrosis in DKD. Cell death is intricately linked to DKD progression, with ferroptosis—a newly identified form of regulated cell death marked by iron overload—playing a crucial role. Current pharmacological interventions that improve renal outcomes in DKD can inhibit ferroptosis in renal cells to varying extents. Thus, targeted ferroptosis inhibition represents a promising therapeutic approach for DKD. Ferroptosis bears a complex interrelationship with inflammatory stress. Innate immune cells, immune molecules, and inflammatory regulatory pathways modulate key processes of ferroptosis, including iron overload, antioxidant system dysfunction, and lipid peroxide accumulation. Conversely, ferroptosis can influence the immunological efficacy of inflammatory cells, release immune-related molecules, and engage in critical inflammatory signaling pathways. Both resident and inflammatory cells in the kidney are susceptible to ferroptosis and inflammatory responses. Currently, the understanding of the relationship between intrinsic immunity and DKD has advanced, but research on the involvement of ferroptosis in DKD is primarily focused on key pathways such as GPx4. Besides, the interplay between ferroptosis and intrinsic immunity remains largely speculative and requires further exploration through well-designed fundamental research in the future. As our understanding of DKD pathogenesis deepens, novel therapeutics targeting inflammation and ferroptosis are actively being explored. Nevertheless, the treatment and management of DKD remain challenging and prolonged, necessitating collaborative efforts across multiple disciplines.
